# Tuning magnetic properties for domain wall pinning via localized metal diffusion

**DOI:** 10.1038/s41598-017-16335-z

**Published:** 2017-11-24

**Authors:** T. L. Jin, M. Ranjbar, S. K. He, W. C. Law, T. J. Zhou, W. S. Lew, X. X. Liu, S. N. Piramanayagam

**Affiliations:** 1 Division of Physics and Applied Physics, School of Physical and Mathematical Sciences, Nanyang Technological University, Singapore, 637371 Singapore; 2 Data Storage Institute, A*STAR (Agency for Science, Technology and Research), 2 Fusionopolis Way, #08-01 Innovis, Singapore, 138634 Singapore; 3 Department of Electrical and Computer Engineering, Shinshu University, Wakasato 4-17-1, Nagano, 380-8553 Japan

## Abstract

Precise control of domain wall displacement in nanowires is essential for application in domain wall based memory and logic devices. Currently, domain walls are pinned by creating topographical notches fabricated by lithography. In this paper, we propose localized diffusion of non-magnetic metal into ferromagnetic nanowires by annealing induced mixing as a non-topographical approach to form pinning sites. As a first step to prove this new approach, magnetodynamic properties of permalloy (Ni_80_Fe_20_) films coated with different capping layers such as Ta, Cr, Cu and Ru were investigated. Ferromagnetic resonance (FMR), and anisotropy magnetoresistance (AMR) measurements were carried out after annealing the samples at different temperatures (*T*_*an*_). The saturation magnetization of Ni_80_Fe_20_ film decreased, and damping constant increased with *T*_*an*_. X-Ray photoelectron spectroscopy results confirmed increased diffusion of Cr into the middle of Ni_80_Fe_20_ layers with *T*_*an*_. The resistance vs magnetic field measurements on nanowires showed intriguing results.

## Introduction

**Figure 1:**
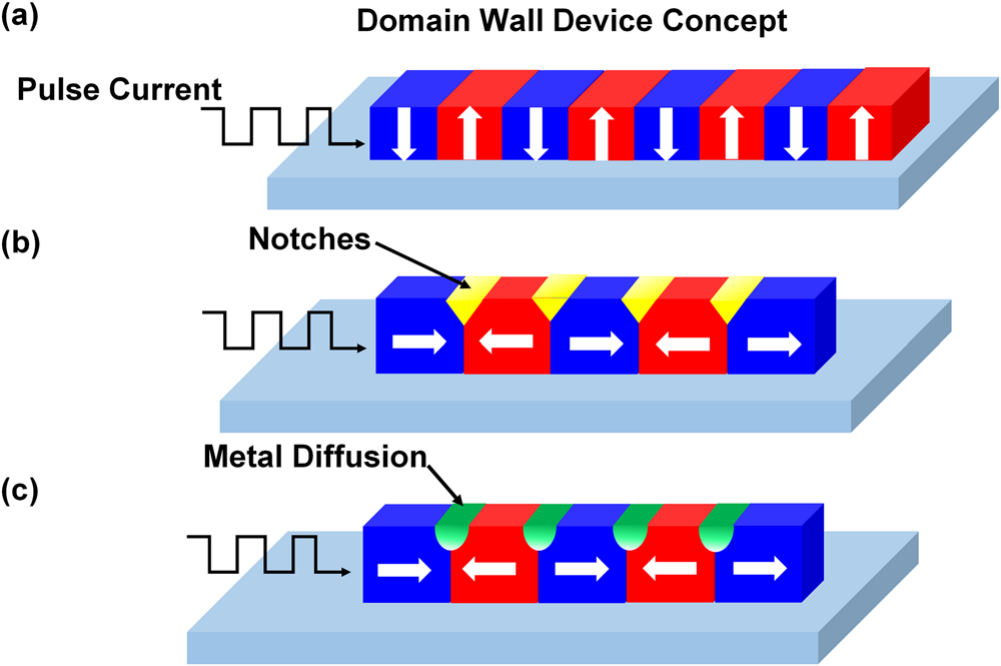
Illustration of domain wall device concept and the control of domain wall propagation using different structures. (**a**) Information stored in nanowire based on domain orientation and an illustration of domain wall movement by pulse current for reading (writing) information. (**b**) Illustration of controlling domain wall propagation using notches. (**c**) Schematic representation of the proposed method to control domain wall motion using metal diffusion.

Domain wall based devices such as racetrack memory have been proposed as promising candidates for high-density, non-volatile information storage with a low energy consumption^1–9^. Such devices are also considered as 3-terminal memory devices at a higher level of memory-storage hierarchy. Information in these devices are stored in the directions of domain magnetization and read and written by moving domain walls. Since the domain wall motion is based on spintronics principles, the reading and writing process do not require mechanical rotation as in hard disk drives^8–10^. Figure 1(a) shows the simplified schematic diagram of domain wall memory that stores information in nanowire based on domain orientation and domain wall movement by pulse current for reading (writing) information. Domain wall motion along the nanowire, driven by in-plane current, has been investigated tremendously^9,11–13^. The theory for spin-transfer torque was reported by Berger and Slonczewski in 1996. Independently, they pointed out that a spin polarized current is generated when an electric current that goes through the ferromagnetic layer transfers the spin angular momentum to local magnetic moment via electron exchange interaction. This exerts a torque on the local magnetization, resulting in domain wall motion^14–18^. In earlier research work, the torque exerted by in-plane current named as spin transfer torque (STT), drove domain wall motion in the opposite direction of electric current flow, and is often considered as an effective field^19,20^. In those cases, the velocity of domain wall was just 100 m/s^19,20^. In the recent years, faster domain wall motion up to 400 m/s, has been observed in perpendicular magnetic anisotropy (PMA) material using pure spin polarized current^12,21,22^. In such structures, the pure spin polarized current originates from the electric current in heavy metal due to strong spin orbit coupling and Rashba effect, which is named as spin orbit torque (SOT)^23,24^. The high speed of domain wall motion also relate to the chiral domain wall structure in PMA material that is formed by interfacial Dzyaloshinskii-Doriya interaction (DMI)^25^. Recently, the speed of domain wall 750 m/s was reported in synthetic anti-ferromagnetic structure driven by SOT^26^.

Spin polarized current gives a high speed performance of domain wall devices. However, for reliable operation of domain wall devices, the other key issue is the control of domain wall motion by pinning domain walls uniformly^9,27–29^. In the absence of pinning sites, the domain walls may be swept rapidly without precise control. In general cases, the pinning sites for domain wall devices are fabricated by complicated lithography process, like creation of notches and zigzag patterns in the ferromagnetic nanowire^6,29–31^. Moreover, the pinning effect could be non-uniform due to the shape and size variations, as shown in Fig. 1(b). Tuning the properties of nanowire locally to pin domain wall at precise position is more efficient for domain wall devices. Exchange bias has been proposed as one such method^32^. Several other methods to tune the properties of ferromagnetic, like focused Ga^+^ ion irradiation on film, non-magnetic metal doped in ferromagnetic film by ion implantation or co-deposition have also been reported^33–37^. Here, we propose an alternative method for controlling domain wall displacement by tuning the composition of ferromagnetic material locally with annealing to stabilize domain walls, as shown in Fig. 1(c). Experimental investigations were carried out to understand the effect of composition on the properties of Ni_80_Fe_20_ film. Diffusion of various metals including Ta, Cr, Cu and Ru, which are widely used in memory industry, was achieved at the interface through annealing under certain temperature. The Ni_80_Fe_20_ devices with cross pinning sites have been fabricated to show that this method could be useful in domain wall pinning.

## Experiments

In order to find out if composition in magnetic films can be controlled by annealing induced mixing and if the properties can be tailored, 10 nm thick Ni_80_Fe_20_ thin films were sputtered with different metallic capping layers. The film stacks of the type Si/(SiO_2_)/Ni_80_Fe_20_ (10 nm)/X (5 nm), were deposited by dc magnetron sputtering. Here, X refers to the capping metallic layers such Ta, Cr, Cu and Ru. Permalloy film without capping layer was also prepared as reference. After deposition, Ni_80_Fe_20_ films with capping layer were annealed for 1 hour at different annealing temperatures, *T*_*an*_, from 100 °C to 400 °C in vacuum. Alternating Gradient Force Magnetometer (AGFM), and broadband Ferromagnetic resonance (FMR) spectroscopy measurements were used to obtain the magnetic properties at different *T*_*an*_.

## Results and Discussion

**Figure 2:**
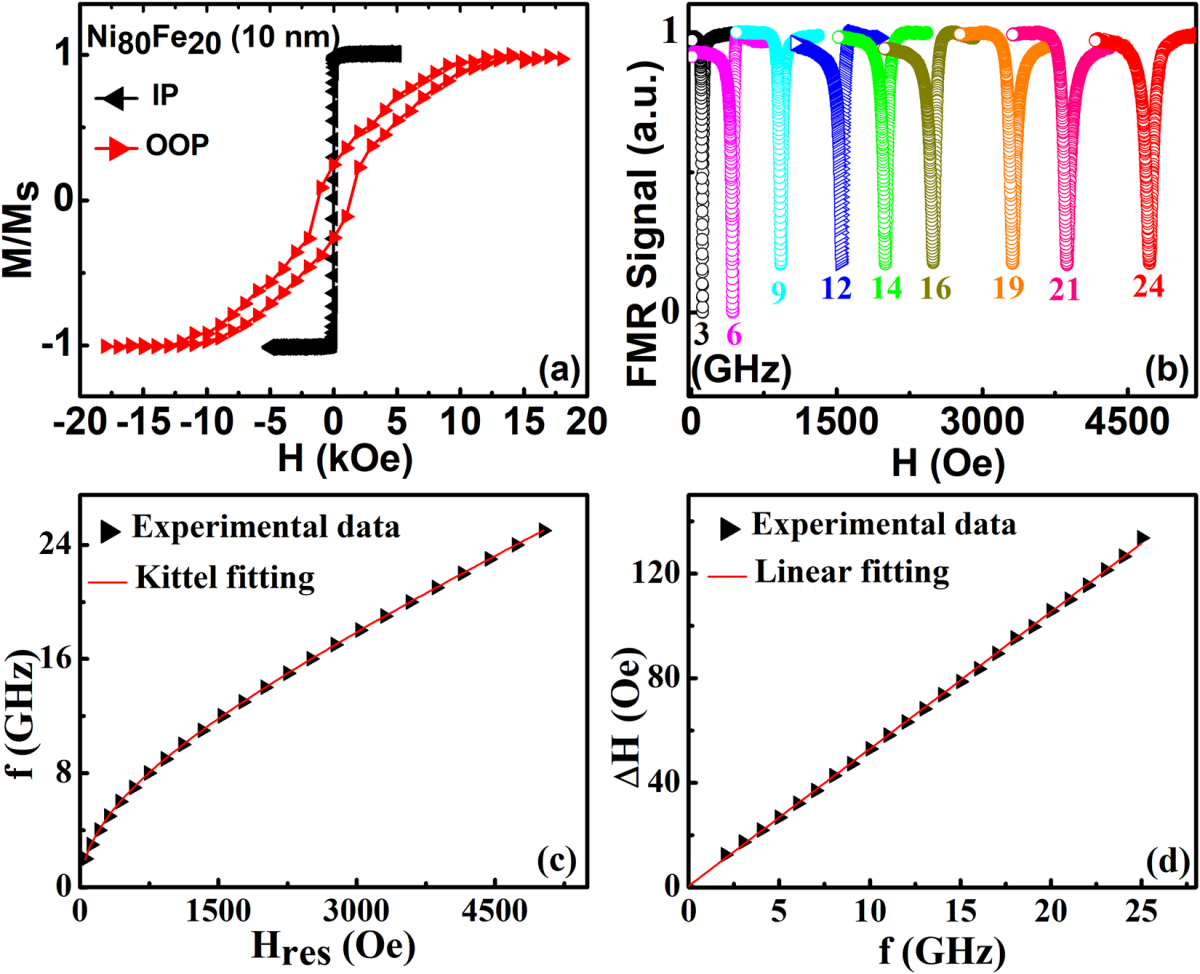
(**a**) The normalized hysteresis loops of Ni_80_Fe_20_ along in plane (IP) direction and out of plane (OOP) directions. (**b**) The FMR signal for Ni_80_Fe_20_ thin film with thickness of 10 nm with magnetic field applied parallel to sample plane. (**c**) Resonance frequency as a function of resonance field and (**d**) FMR linewidths as a function of resonance frequency.

Normalized magnetic hysteresis loops (M-H) of pure Ni_80_Fe_20_ (NiFe) thin film, measured along in plane (IP) and out of plane (OOP) directions, are shown in Fig. 2(a). The M-H loop shows well defined in-plane anisotropy of Ni_80_Fe_20_ film, with a low coercivity of about 15 Oe. In order to study quantitatively the magnetodynamics properties of films, broadband ferromagnetic resonance (FMR) measurements were carried out at room temperature. Figure 2(b–d) shows various steps of FMR linewidth measurements for pure Ni_80_Fe_20_ film, supporting the quality of the film and the measurement. The saturation magnetization is 726 ± 2 emu/cc. The Gilbert damping constant is small for Ni_80_Fe_20_ film, about 0.0077 ± 2 × 10^−4^, and is consistent with the values reported in literature^38,39^.

**Figure 3:**
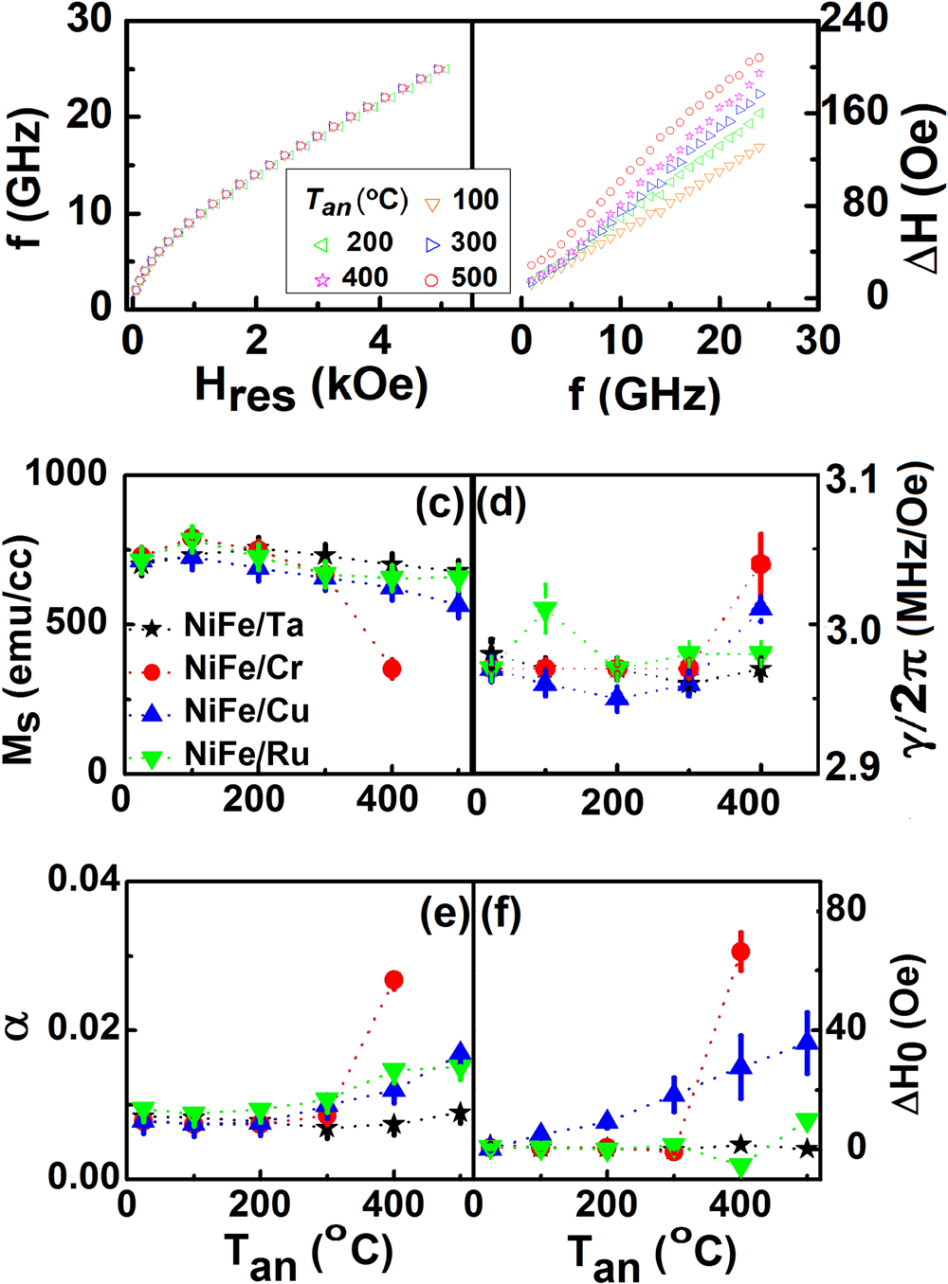
Ni_80_Fe_20_ without capping layer at different annealing temperatures (*T*_*an*_), resonance frequency as a function of resonance field (**a**) and FMR half linewidths as a function of resonance frequency (**b**). Magnetic properties of Ni_80_Fe_20_/X (NiFe/X = Ta, Cr, Cu, Ru) at different annealing temperatures (*T*_*an*_). The effective saturation magnetization (**c**), Gyromagnetic ratio (**d**), damping constant (**e**) and inhomogeneous broadening (**f**).

Standard properties shown in Fig. 2, verified the quality of the prepared permalloy films. As a next step, the static and dynamic properties of Ni_80_Fe_20_ film samples without capping and with different metallic capping layers annealed at different *T*_*an*_ from 100 °C to 400 °C were studied by measuring normalized M-H loops and FMR spectral signal. The FMR spectral signal was obtained by sweeping the magnetic field, which is parallel to film direction, with varying frequency from 2 GHz to 25 GHz. In this configuration, the resonance frequency and applied field follow the Kittel equation relation and the half linewidth of the resonances peak was also measured as a function of frequency. Figures 3(a) and (b) show the resonance frequency vs applied field and the half linewidth vs resonance frequency of Ni_80_Fe_20_ film without capping layer annealed at different *T*_*an*_. To extract the saturation magnetization (*M*_*s*_) of Ni_80_Fe_20_ film samples after annealing, the Kittel equation was fitted to the data as shown in Fig. 3(a). *M*_*s*_ did not show much change after annealing. Even at higher *T*_*an*_ = 400 °C, the value of *M*_*s*_ was maintained at 723 ± 3 emu/cc. But the damping constant, obtained by fitting the linewidth with resonance frequency in Fig. 3(b) (Refer to the Method part), increased after annealing, going up to 0.0122 ± 2 × 10^−4^ at *T*_*an*_ = 400 °C^39,40^. The increase in damping is probably due to the diffusion of oxygen into NiFe from substrate Si (SiO_2_) arising from an increase in the magnon scattering^41^. The damping constant showed a remarkably high value of 0.0185 ± 3 × 10^−4^ at *T*_*an*_ = 500 °C. It is well known that the depinning field increases with the damping constant^42^. As a result, a higher current is required to drive domain wall motion, which is not desired. Therefore, in the subsequent experiments, we fixed the maximum annealing temperature to be 400 °C.

The magnetic properties of NiFe with different capping layer, annealed at different temperatures, are shown in Fig. 3(c) to (f). Saturation magnetization (*M*_*s*_) decreases with increasing *T*_*an*_ from 100 °C to 400 °C for all metallic capping layer as shown in Fig. 3(c). The decrease has been the most significant in the case of Cr, where the effective magnetization *M*_*s*_ drops down to 350 ± 8 emu/cc at 400 °C. In the case of Cu capping layer, *M*_*s*_ decreases from 720 ± 3 emu/cc to 624 ± 5 emu/cc with increasing *T*_*an*_ to 400 °C, while for Ta and Ru, magnetization decreases at a much slower rate. We also found beyond 200 °C, saturation field along the perpendicular direction increases steadily with *T*_*an*_. Figure 3(d–f) show the magnetodynamic properties of Ni_80_Fe_20_/X such as Gyromagnetic ratio (d), damping constant (e) and inhomogeneous broadening (f) at different *T*_*an*_. The Gyromagnetic ratio (*γ/2π*) remained at about 2.96 MHz/Oe for most all the samples. For Cu and Cr capping layer, it increases to 3.01 and 3.04 (MHz/Oe) at *T*_*an*_ = 400 °C respectively. The damping constant of Ni_80_Fe_20_ increased with *T*_*an*_ for all capping layers^33,35,36^. Among all the elements investigated, Cr showed the strongest effect and Ta capping layer had the least effect. The damping constant of Ni_80_Fe_20_ is not influenced much for *T*_*an*_ < 300 °C. However, for Cr, an increase in *α* by 246% at *T*_*an*_ = 400 °C was observed. For Cu capping layer, the damping constant of the annealed films increased to 0.01198 ± 3 × 10^−5^ at *T*_*an*_ = 400 °C^43^. The inhomogeneous broadening value is small for all capping layers at room temperature, and it shows the increasing trend for Cr and Cu capping layers, increasing to 66 Oe and 28 Oe respectively with *T*_*an*_. However, it does not show much change for Ta and Ru capping layers. Anisotropy field was very small as expected for all Ni_80_Fe_20_/X films and it did not show significant changes after annealing.

The investigations discussed so far indicate that the metal diffusion causes changes in the properties of Ni_80_Fe_20_ film and among several elements, Cr has the strongest effect both in static (*M*_*s*_) and dynamic properties (*α*). It is worthwhile to compare the properties with those reported in literature^35,43–46^. Ruiz-Calaforra *et al*., have studied the effect of metal capping layers adjacent to NiFe layer. Some of their samples (9–11 nm thickness of NiFe) are similar to our samples at room temperature. In their samples, the presence of a Pt layer enhances the damping constant significantly due to spin pumping. In comparison, Ru enhances the damping constant to a larger extent than Cr. A similar trend in this study was also observed at room temperature. However, absolute values and the changes in the damping constant are smaller in our samples due to less noise in our FMR data and probably the nature of deposition process of our samples. In our samples, Cr capping layers did not cause a significant change in the damping constant at room temperature. However, it causes a large increase in damping constant after annealing. From this comparison, the increase in damping constant is mainly attributed to the annealing induced mixing. Faulkner *et al*.^36^, have studied the influence of Cr doping by ion implantation in NiFe. They too observe a similar increase in damping constant, a decrease in *M*_*s*_ and anisotropy energy (*K*_*u*_). It is well known that the addition of Cr causes decrease of magnetization in ferromagnetic systems. Cr spins are believed to couple antiferromagnetically to the spins of host atoms and as a result, our samples too show a decrease of *M*_*s*_.

**Figure 4:**
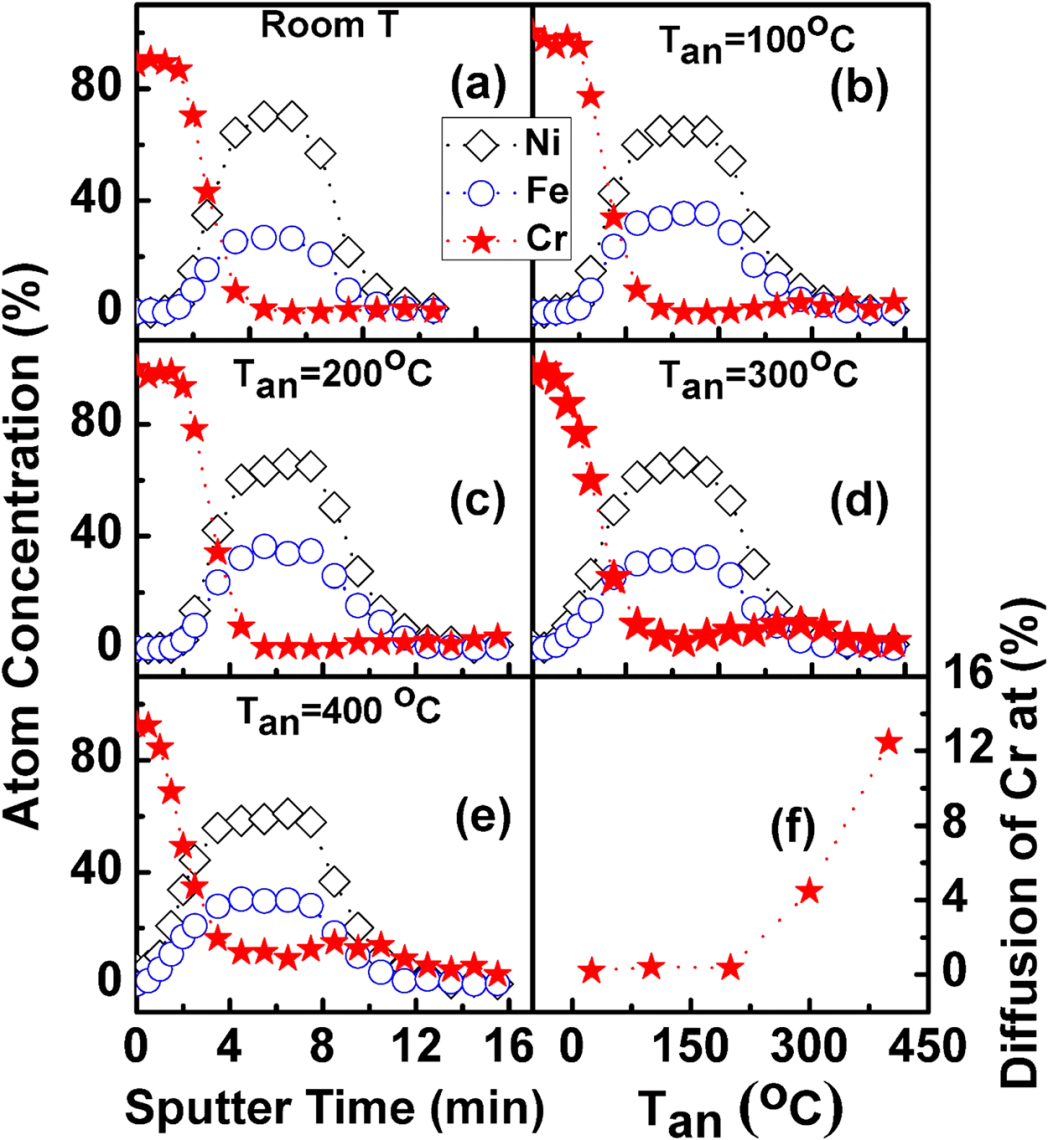
XPS analysis for Cr diffusion effect vs the *T*_*an*_ from 100 °C to 400 °C ((**a**) to (**e**)) and (**f**) summarizes the concentration of Cr diffusion into Ni_80_Fe_20_ layer with the *T*_*an*_ taking the diffusion value of Cr at 7 minutes sputter etching time.

In order to understand the diffusion of Cr in Ni_80_Fe_20_ film further, X-ray photoelectron spectroscopy (XPS) was carried out. Figure 4(a) to (e) show the Cr diffusion effect with the different *T*_*an*_. It can be seen that the Cr diffusion is less for *T*_*an*_ below 300 °C, but shoots up for *T*_*an*_ above 300 °C. Figure 4(f) summarizes the concentration of Cr, closer to the bottom of Ni_80_Fe_20_ layer (7 minutes sputter etching time), for different *T*_*an*_. The changes in saturation magnetization *M*_*s*_, damping constant and the other properties can be explained to be arising as a result of Cr diffusion, which is significantly stronger at higher temperatures.

**Figure 5:**
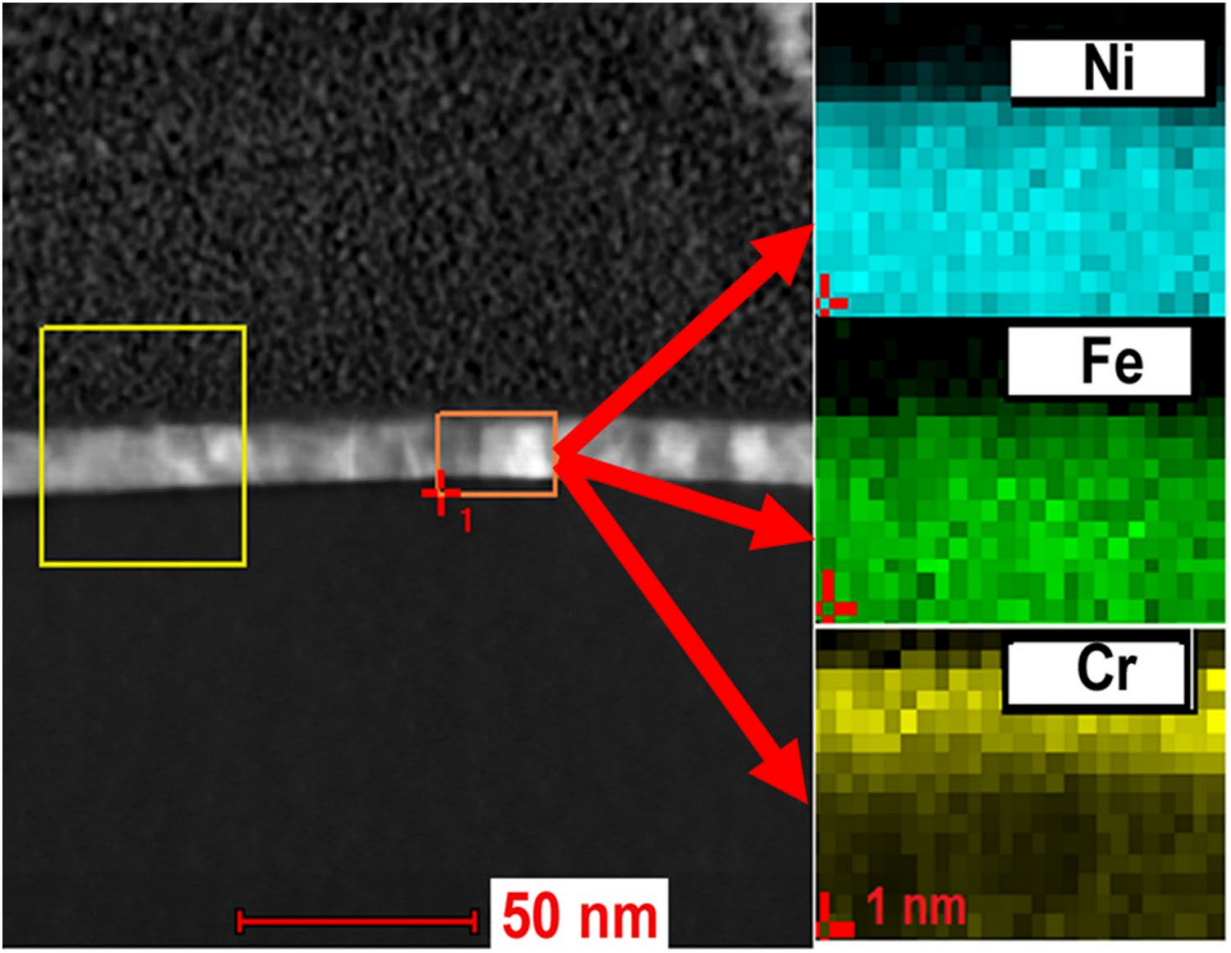
TEM cross configuration and the EDX analysis pattern result at the square area shown as the arrow. From top to bottom, the images show the Ni, Fe and Cr the quantitative analysis.

**Figure 6:**
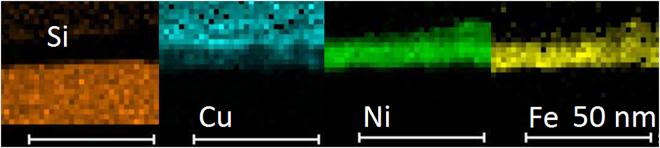
Elemental map of Si, Cu, Ni and Fe in Si substrate/NiFe/Cu structures.

In order to understand if the elements diffuse in to NiFe uniformly and form solid solutions or if they form clusters, we also carried out elemental analysis of the interfaces. For this purpose, energy dispersive X-ray spectroscopy was carried out by using Transmission Electron Microscopy (TEM) equipment. Figure 5 shows the TEM cross section configuration of NiFe with Cr capping layer annealed at 400 °C and Energy Dispersive X-ray (EDX) Spectroscopy analysis images. The results indicate that the diffusion of Cr into NiFe layer by annealing is uniform and hence a solid solution. Different elements may behave differently and their behavior may, to some extent, be predicted from the phase diagrams^47–49^. It is well known that Cu does not mix well with Fe and hence, we should see different results. Figure 6 shows the EDX analysis of elemental concentration of Si (substrate), Ni, Fe and Cu. It can be seen that the copper atoms do not diffuse so uniformly like Cr atoms.

**Figure 7:**
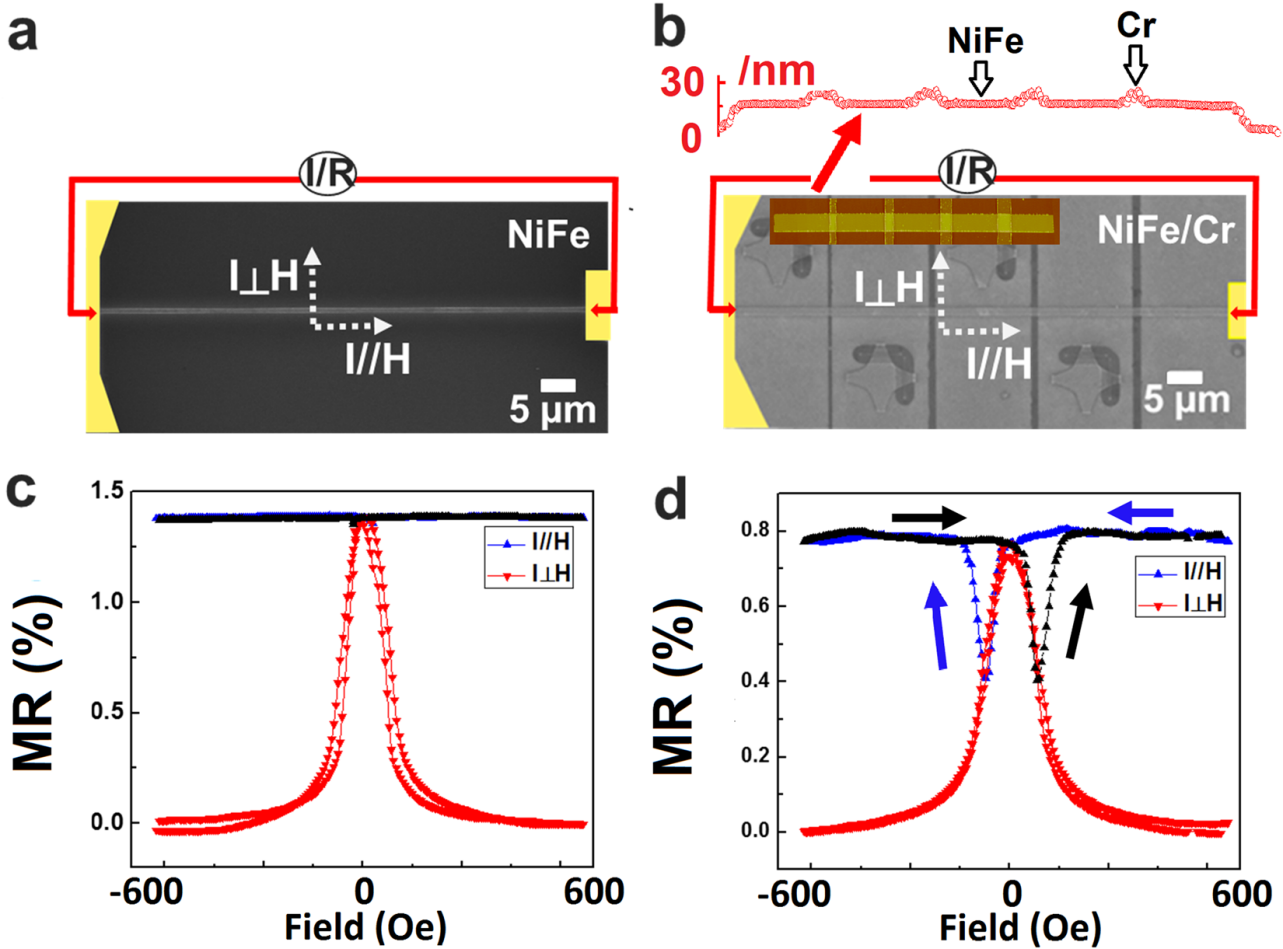
Schematic representation of NiFe magnetic wire without Cr pinning cross site (**a**) and with Cr pinning sites (**b**). The insert figure is AFM image of NiFe wire with Cr cross wires. The resistance ratio of NiFe with Cr pining bars (**c**) and without Cr pinning sites (**d**) measured by applying in-plane magnetic field along and perpendicular to NiFe wire direction.

In order to prove that the local modification is effective in stabilizing the domain walls, NiFe magnetic wires with 1 µm wide and 100 µm long were fabricated by Electron-beam lithography (EBL) and argon ion milling. A Ta seedlayer was used to prevent the diffusion of oxygen from Si substrate and to improve the adhesion^50^. After the NiFe wires were fabricated, Cr cross lines were fabricated by EBL, followed by 5 nm thick Cr deposition. The NiFe magnetic wire was annealed in vacuum chamber at 400 °C atmosphere before all the electrode fabrication. A top view micrograph of the magnetic wire is shown in Fig. 7(b). The inset in Fig. 7(b) is the line-scan of Atomic Force Microscopy (AFM) images of the NiFe wire with Cr wires, and the height profile shows that the Cr is on top of the NiFe wire. The NiFe magnetic wire without Cr pinning cross lines also was prepared as reference sample using EBL, as shown in Fig. 7(a).

NiFe without Cr magnetic wire shows typical properties in AMR measurements. When field was applied parallel to the current direction, a high resistance was observed and for field perpendicular to the current direction, a drop in resistance was observed at high fields as shown in Fig. 7(c). A high magnetic field perpendicular to the nanowire orients the magnetization in perpendicular direction to the current and hence such a drop is observed. In the case of magnetic field along the nanowire direction, the change in resistance is not significant, as the domains are swept rapidly and hence the magnetization is always parallel or antiparallel to the current direction.

In the case of NiFe wires with Cr pinning bars, the results for the case of current perpendicular to the field are similar. However, the observed results for the case of current parallel to the magnetic field are different and interesting. Two obvious dips are observed in the R-H curves as shown as Fig. 7(d). These dips were observed at fields, as high as −100 Oe, which are much larger than the coercivity. Although such a dip could arise as a result of domain wall pinning, the significantly large magnitude of the dip warrants further investigation to understand the origin of such a dip. When a large magnetic field (600 Oe) along the nanowire was applied, the magnetization is saturated and parallel to the current, and the resistance was larger again.

## Conclusion

To summarize, a novel domain wall pinning method using compositional modifications is proposed and the materials level investigations are carried out experimentally in detail. Investigations at thin-film level indicate that the diffusion caused by annealing is useful to form domain wall pinning sites. Diffusion of Cr proves to be more effective in comparison to other elements such as Ta, Cu and Ru in changing the magnetic and magnetodynamic properties. Domain wall devices were fabricated and investigated using resistance measurements as a gauge of domain wall pinning. Compared to permalloy wires without Cr-diffused pinning sites, permalloy wires with Cr-diffused pinning sites exhibited different properties. Although annealing is investigated in this study, compositional modification can also be carried out using techniques such as ion-implantation. For much higher density operation, materials with a perpendicular magnetic anisotropy may be used, although this study only uses permalloy^29,50^. The method proposed in this study is promising to stabilize and control domain wall propagation in domain wall memory devices.

## Methods

### Sample fabrication

All the Ni_80_Fe_20_ samples were deposited on silicon substrates covered by 500 μm thermal SiO_2_ layer via DC magnetron sputtering in AJA sputtering system at room temperature. The base pressure of the main chamber was superior to 1.1 × 10^−7^ Torr. The argon gas pressure for Ni_80_Fe_20_ layer was fixed at 2 mTorr and the deposition rate was 0.12 nm s^−1^ fired at the DC power of 40 W. Different capping layers Ta, Cr, Cu, and Ru were deposited at an argon pressure of 3 mTorr respectively. 10 nm thick Ni_80_Fe_20_ thin film with different non-magnetic metal capping layers (Ni_80_Fe_20_/X = Ta, Cr, Cu, and Ru) of 5 nm samples were fabricated. After deposition, Ni_80_Fe_20_ films with capping layer were annealed for 1 hour at different *T*_*an*_ from 100 °C to 400 °C in vacuum condition with base pressure below 5 × 10^−7^ Torr.

Thickness calibration is based on measuring the thickness of the stepped film by using Atomic Force Microscopy. The stepped film was fabricated by drawing straight line using a marker pen, followed by washing off in Isopropyl alcohol.

The fabrication of NiFe and Cr wires was made by E-beam lithography (EBL). The first layer of resist over NiFe wire was exposed under 20 kV voltage and 7 µm aperture. After exposure, Ar ion-milling was done to eliminate the unwanted film. The second layer of resist over NiFe wires was exposed under 10 kV voltage and 30 µm aperture. After developing, Cr was deposited in the holes of the second layer of resist. The device in vacuum chamber was annealed at 400 °C for one hour to make Cr diffusion into the NiFe wire at the cross section to form pinning sites. After this, fabrication of the electrode pad was carried out by exposing the resist under 20 kV and 120 µm aperture.

### Sample Characterization

Normalized magnetic hysteresis loops (M-H) of Ni_80_Fe_20_ thin film along in plane (IP) and out of plane (OOP) directions were carried out at room temperature with the magnetic sweeping from −20 kOe to 20 kOe. Broadband ferromagnetic resonance (FMR) measurements were carried out using a Keysight PNA N5222A vector network analyzer with a 117 µm width coplanar waveguide at room temperature to study quantitatively the magnetic static and dynamics properties of films, including saturation magnetization (*M*_*s*_), in-plane anisotropy field (H_k_), Gyromagnetic ratio (*γ*), damping constant (α) and inhomogeneous broadening (∆H_0_). The microwave frequency was varied from 2 GHz to 25 GHz and the magnetic field was applied parallel to sample plane. The ferromagnetic resonance frequency $f$, in the applied field is determined by the following equation:

1 $${f}=\frac{\gamma }{2\pi }{[({H}_{appl}+{H}_{k})\times ({H}_{appl}+{H}_{k}+{M}_{s})]}^{\frac{1}{2}}$$

The damping relation with frequency dependent FMR linewidths:

2 $${\rm{\Delta }}H=\frac{4\pi }{\gamma }\alpha f+{\rm{\Delta }}{H}_{0}.$$
